# Prevalence and Associated Factors of Hypertension Among Diabetic Individuals in the Sylhet Region of Bangladesh

**DOI:** 10.1155/ijhy/9276454

**Published:** 2026-09-07

**Authors:** Abu Taher, Nurshad Ali

**Affiliations:** ^1^ Department of Biochemistry and Molecular Biology, Shahjalal University of Science and Technology, Sylhet 3114, Bangladesh, sust.edu

**Keywords:** adults, bangladesh, diabetes, hypertension, risk factors

## Abstract

**Background:**

Hypertension commonly coexists with diabetes and substantially increases the risk of cardiovascular and renal complications. However, data on the prevalence of hypertension and its associated factors among people with diabetes in Bangladesh remain limited. This study aimed to determine the prevalence of hypertension and identify factors associated with hypertension among adults with diabetes in the Sylhet region of Bangladesh.

**Methods:**

A total of 295 adults with diabetes were enrolled in this cross‐sectional study. Data on anthropometric measurements, lifestyle factors, and sociodemographic characteristics were collected using a structured questionnaire and a review of medical records. Diabetes and hypertension were defined according to standard guidelines. Blood glucose and lipid profile parameters were measured using standard colorimetric methods. Multivariable logistic regression analysis was performed to identify factors associated with hypertension.

**Results:**

The overall prevalence of hypertension was 61.4% (181/295). Hypertension prevalence increased with age and adiposity, reaching 69.4% among participants aged > 55 years and 70.0% among those with obesity. In the multivariable logistic regression analysis, participants aged > 55 years had higher odds of hypertension than those aged 30–40 years (AOR = 2.09, 95% CI: 1.10–3.97). Compared with participants with normal BMI, overweight (AOR = 1.82, 95% CI: 1.03–3.20) and obesity (AOR = 2.46, 95% CI: 1.24–4.88) were significantly associated with hypertension. Abdominal obesity was also independently associated with hypertension (AOR = 2.57, 95% CI: 1.41–4.65). No significant associations were observed for sex, education level, dyslipidemia, lipid parameters, physical activity, or smoking status.

**Conclusions:**

Hypertension was highly prevalent among adults with diabetes in the Sylhet region of Bangladesh. Older age and both general and abdominal obesity were independently associated with hypertension, underscoring the importance of regular blood pressure screening and comprehensive weight management as integral components of diabetes care.

## 1. Introduction

Hypertension and diabetes are significant noncommunicable diseases worldwide. Their coexistence poses a major challenge for public health sectors, especially in low‐ and middle‐income countries [1]. When hypertension occurs alongside diabetes, it greatly increases the risk of serious complications such as stroke, coronary artery disease, kidney disease, and eye disease [2]. Studies show that people with diabetes and high blood pressure have much worse health outcomes than those with either condition alone [2, 3].

In South Asia, the burden of diabetes and its related complications has increased more rapidly than in many other regions of the world [4]. This trend indicates the high vulnerability of the South Asian population, coupled with rapid changes in lifestyle and environment [4]. Recent studies from the region indicate a notable increase in diabetes cases over the past 2 decades [5]. Urban dwellers and older individuals seem to be particularly affected. There is a well‐established connection between diabetes and hypertension within South Asian populations, with numerous studies showing that many people with diabetes also struggle with high blood pressure [4, 6]. This trend significantly elevates the cardiovascular risk for the population.

In Bangladesh, the prevalence of hypertension among the adult population is already high and continues to rise. According to the Bangladesh Demographic and Health Survey (BDHS) 2017–18, 27.4% (95% CI: 26.5%–28.5%) of adults aged 18 years and older had hypertension [7, 8]. Moreover, diabetes and hypertension often occur together. Analysis of BDHS data found that people with diabetes have 1.54 times higher odds of having hypertension compared to those without diabetes (adjusted odds ratio [AOR]: 1.54; 95% CI: 1.31–1.83) [8]. A hospital‐based study among individuals with Type 2 diabetes in Bangladesh reported a hypertension prevalence of 67.2% [9]. These findings highlight the importance of the issue and the necessity for focused research.

In the regional context of northeastern Bangladesh, especially in the Sylhet region, there are limited data on the prevalence and causes of hypertension among people with diabetes. Given the changing health landscape in Bangladesh, marked by rising obesity, sedentary lifestyles, urbanization, and changes in diet, the risk of hypertension in diabetic adults may be especially high in these areas. Research shows that older age, being overweight or obese, having diabetes for a long time, a lack of physical activity, and having chronic kidney disease are key risk factors for hypertension in people with diabetes [9].

It is therefore crucial to examine the burden and factors associated with hypertension among individuals with diabetes at the local level to inform targeted interventions. Understanding the sociodemographic, behavioral, and clinical factors associated with hypertension in this population may help healthcare providers and policymakers develop effective strategies for its screening, prevention, and management. Therefore, this cross‐sectional study aimed to (i) estimate the prevalence of hypertension among adults with diabetes in the Sylhet region of Bangladesh and (ii) identify factors associated with hypertension in this population.

## 2. Methods

### 2.1. Study Population

This cross‐sectional study was conducted from November 2019 to October 2022 in the Department of Biochemistry and Molecular Biology, Shahjalal University of Science and Technology (SUST), Sylhet, Bangladesh. Adult participants with diabetes residing in the Sylhet region were recruited using a nonprobability, convenience‐based sampling approach. Eligible individuals were invited to participate through direct recruitment. A total of 295 participants aged 30–80 years were included in the study. Both men and women were eligible for participation. Individuals were excluded if they were pregnant or breastfeeding or had a history of drug addiction or alcohol use, liver or kidney disease, cancer, or infectious disease. Participants who declined participation or had incomplete information required for the study were also excluded. Both sexes were invited using the same recruitment procedures; however, substantially fewer women volunteered to participate, resulting in an imbalanced sex distribution.

The study protocol was approved by the Internal Ethics Committee of the Department of Biochemistry and Molecular Biology, SUST (Reference No. 02/BMB/2019). All study procedures were conducted in accordance with relevant ethical guidelines and regulations. Written informed consent was obtained from all participants before their inclusion in the study.

### 2.2. Data Collection

Anthropometric measurements included body weight, height, waist circumference (WC), and hip circumference (HC). Measurements were obtained by trained personnel following standardized procedures described in previous studies [10–14]. Body mass index (BMI) was calculated as weight in kilograms divided by height in meters squared. Systolic and diastolic blood pressure were measured using an Omron M10 digital sphygmomanometer (Omron, Tokyo, Japan). Information on diabetes status, hypertension, other chronic conditions, and relevant lifestyle characteristics was collected using a structured questionnaire.

### 2.3. Blood Collection and Laboratory Analysis

Following an overnight fast of 10–12 h, 4 mL of venous blood was collected from each participant in the morning by trained personnel. Blood specimens were immediately transferred under cooled conditions to the laboratory, where serum was separated by centrifugation at 4400 rpm for 10 min using a Sorvall ST 8R centrifuge (Thermo Scientific, Germany). Serum aliquots were stored at −80°C until biochemical analyses. Fasting blood glucose, serum uric acid, creatinine, total cholesterol, triglycerides, HDL‐C, and LDL‐C were quantified using commercially available Human Diagnostic kits (Germany) and a Humalyzer 3000 biochemical analyzer (USA), following the manufacturers’ instructions.

### 2.4. Diagnostic Criteria

Hypertension was classified according to the criteria of the Seventh Report of the Joint National Committee, defined as SBP ≥ 140 mmHg and/or DBP ≥ 90 mmHg, or current use of antihypertensive medication [15]. Diabetes was defined according to the American Diabetes Association criteria as fasting plasma glucose ≥ 126 mg/dL, random (non‐fasting) plasma glucose ≥200 mg/dL, or current use of glucose‐lowering medication [16]. Physical activity was assessed using the WHO Global Physical Activity Questionnaire [17]. Physical activity was classified into three categories: low, moderate, and vigorous based on the level of effort involved. Low activity includes office work and light housework. Moderate activity includes general walking, swimming, and light cleaning. Vigorous activity involves tasks such as lifting, carrying, jogging, and playing sports.

### 2.5. Statistical Analysis

Statistical analyses were performed using IBM SPSS Statistics Version 25.0 (SPSS Inc., Chicago, IL, USA). Continuous variables are presented as mean ± standard deviation, whereas categorical variables are summarized as frequencies and percentages. Differences between groups were assessed using independent‐samples *t*‐tests for continuous variables and Pearson’s chi‐square tests for categorical variables. Multivariable logistic regression was used to examine factors independently associated with hypertension. Variables considered clinically relevant or showing evidence of association in preliminary analyses were entered into the multivariable model. Multicollinearity was evaluated using variance inflation factor and tolerance statistics. AORs with 95% confidence intervals (CIs) were reported, and *p* < 0.05 was considered statistically significant.

## 3. Results

### 3.1. Baseline Characteristics of the Study Subjects

The study included 295 individuals with diabetes, of whom 271 were male and 24 were female (Table 1). The mean age of the study population was 47.3 ± 11.9 years, and the mean BMI was 25.3 ± 3.8 kg/m^2^. Overall, 51.0% of participants were overweight, and 23.8% were obese. Among participants with available WC data, 38.2% had abdominal obesity.

**TABLE 1:** Characteristics of the study participants.

	Total	Male	Female	*p* value
*N*	295	271	24	
Age (years)	47.3 ± 11.9	47.7 ± 11.9	42.7 ± 11.4	0.049
Age groups, *N* (%)				
30–40	96 (32.5)	84 (31)	12 (50)	
41–55	127 (43.1)	118 (43.5)	9 (37.5)	0.125
> 55	72 (24.4)	69 (25.5)	3 (12.5)	
BMI (kg/m^2^)	25.3 ± 3.8	25.1 ± 3.7	27.7 ± 4.7	0.001
BMI group, *N* (%)				
Normal	74 (25.2)	71 (26.3)	3 (12.5)	0.006
Overweight	150 (51)	141 (52.2)	9 (37.5)	
Obesity	70 (23.8)	58 (21.5)	12 (50)	
WC (cm)	86.7 ± 10.1	86.8 ± 10.1	85.9 ± 9.5	0.720
WC groups, *N* (%)				
Normal	139 (61.8)	135 (64.6)	4 (25)	0.002
Abdominal obesity	86 (38.2)	74 (35.4)	12 (75)	
Education level, *N* (%)				
Illiterate	37 (16.2)	35 (16.4)	2 (13.3)	0.003
Primary	49 (21.5)	48 (22.5)	1 (6.7)	
Secondary/higher secondary	70 (30.7)	69 (32.4)	1 (6.7)	
Graduate or above	72 (31.6)	61 (28.6)	11 (73.3)	
FBG (mg/dL)	172.6 ± 78.6	172.1 ± 78.4	178 ± 82.2	0.738
TG (mg/dL)	208.5 ± 132.9	209.9 ± 136	194.6 ± 95.8	0.490
TC (mg/dL)	217.2 ± 92.1	218.6 ± 95	203.1 ± 57.6	0.307
LDL‐C (mg/dL)	143.2 ± 85.8	144.2 ± 88.1	132.9 ± 60.4	0.464
HDL‐C (mg/dL)	35.4 ± 14.9	35.6 ± 15.5	32.6 ± 6.5	0.118
Physical activity, *N* (%)				
Low	57 (25.3)	55 (26.2)	2 (13.3)	0.523
Medium	146 (64.9)	135 (64.3)	11 (73.3)	
Adequate	22 (9.8)	20 (9.5)	2 (13.3)	
Smoking				
No	240 (81.4)	216 (79.7)	24	0.014
Yes	55 (18.6)	55 (20.3)	0	

*Note:* Data are presented as mean ± SD or %. TG, triglycerides; *p* values are derived from the independent sample *t*‐test for continuous variables and the chi‐square test for categorical variables.

Abbreviations: BMI, body mass index; DBP, diastolic blood pressure; HDL‐C, high‐density lipoprotein cholesterol; LDL‐C, low‐density lipoprotein cholesterol; SBP, systolic blood pressure; TC, total cholesterol; WC: waist circumference.

The mean fasting blood glucose, TG, TC, LDL‐C, and HDL‐C levels were 172.6 ± 78.6, 208.5 ± 132.9, 217.2 ± 92.1, 143.2 ± 85.8, and 35.4 ± 14.9 mg/dL, respectively. Most participants reported moderate levels of physical activity (64.9%), while 18.6% reported smoking. Educational attainment varied across the study population, with 31.6% having graduate‐level or higher education. Although some differences in anthropometric and sociodemographic characteristics were observed between male and female participants, these comparisons should be interpreted cautiously because of the small number of female participants.

### 3.2. Prevalence of Hypertension Among the Participants

**FIGURE 1:**
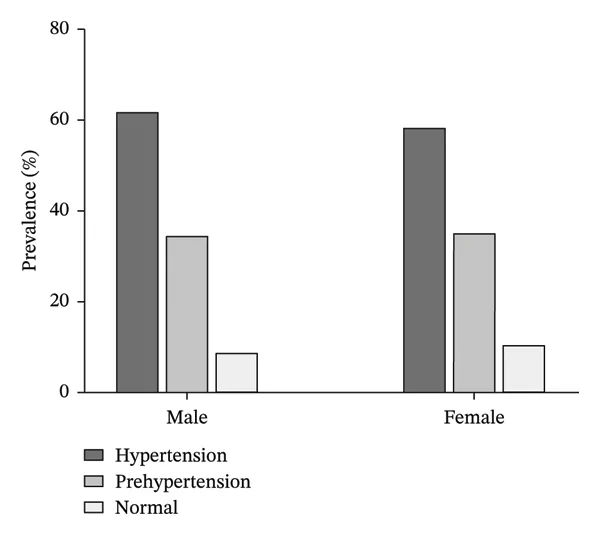
Prevalence of hypertension, prehypertension, and normal blood pressure status among Type 2 diabetic individuals according to gender.

**FIGURE 2:**
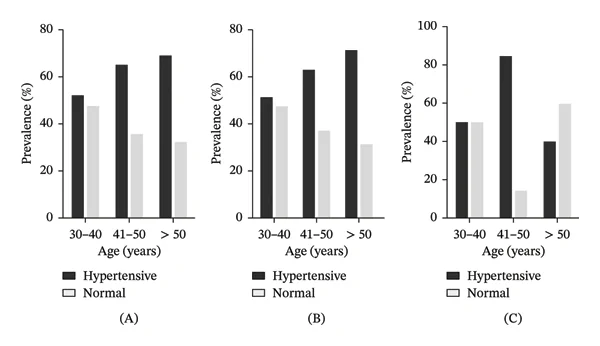
Age‐specific prevalence of hypertension among Type 2 diabetes patients. (A) Overall population, (B) males, and (C) females across different age groups (30–40, 41–50, and > 50 years).

Among the 295 participants with diabetes, the overall prevalence of hypertension was 61.4% (Table 2 and Figure 1). Hypertension prevalence did not differ significantly by sex, occurring in 61.6% of males and 58.3% of females (*p* = 0.751). Although hypertension prevalence increased progressively with age, from 52.1% among participants aged 30–40 years to 63.8% among those aged 41–55 years and 69.4% among those aged > 55 years, this trend did not reach statistical significance (*p* > 0.05) (Figure 2). Hypertension prevalence increased significantly across BMI categories, from 48.6% among participants with normal BMI to 63.3% among those who were overweight and 70.0% among those with obesity (*p* = 0.024). Similarly, hypertension was more prevalent among participants with abdominal obesity than among those with normal WC (75.6% vs. 54.7%, *p* = 0.002). No statistically significant differences in hypertension prevalence were observed according to education level (*p* = 0.420), physical activity (*p* = 0.261), or smoking status (*p* = 0.078).

**TABLE 2:** Prevalence of hypertension among diabetic individuals according to baseline characteristics.

Characteristic	*n* (%)	Hypertension		*p* value
		Yes, *n* (%)	No, *n* (%)	
Overall	295 (100)	181 (61.4)	114 (38.6)	—

*Gender*				
Male	271 (91.9)	167 (61.6)	104 (38.4)	0.751
Female	24 (8.1)	14 (58.3)	10 (41.7)	

*Age groups*				
30–40	96 (32.5)	50 (52.1)	46 (47.9)	0.056
41–55	127 (43.1)	81 (63.8)	46 (36.2)	
> 55	72 (24.4)	50 (69.4)	22 (30.6)	

*BMI group*				
Normal	74 (25.2)	36 (48.6)	38 (51.4)	0.024
Overweight	150 (51.0)	95 (63.3)	55 (36.7)	
Obesity	70 (23.8)	49 (70.0)	21 (30.0)	

*WC groups*				
Normal	139 (61.8)	76 (54.7)	63 (45.3)	0.002
Abdominal obesity	86 (38.2)	65 (75.6)	21 (24.4)	

*Education level*				
Illiterate	37 (16.2)	22 (59.5)	15 (40.5)	0.420
Primary	49 (21.5)	36 (73.5)	13 (26.5)	
Secondary/higher secondary	70 (30.7)	44 (62.9)	26 (37.1)	
Graduate or above	72 (31.6)	43 (59.7)	29 (40.3)	

*Physical activity*				
Low	57 (25.3)	41 (71.9)	16 (28.1)	0.261
Medium	146 (64.9)	87 (59.6)	59 (40.4)	
Adequate	22 (9.8)	14 (63.6)	8 (36.4)	

*Smoking*				
No	240 (81.4)	153 (63.8)	87 (36.2)	0.078
Yes	55 (18.6)	28 (50.9)	27 (49.1)	

*Note:* Data are presented as *n* (%). Percentages in the overall column are calculated using the total number of participants with available data for each variable; percentages in the hypertension and no‐hypertension columns are calculated within the respective groups. *p* values were obtained using Pearson’s chi‐square test.

### 3.3. Potential Factors Associated With Hypertension

Multivariable logistic regression analysis identified several factors significantly associated with hypertension among individuals with diabetes (Table 3). The analysis identified age, BMI, and abdominal obesity as significant factors associated with hypertension among individuals with diabetes. Participants aged > 55 years had approximately twice the adjusted odds of hypertension compared with those aged 30–40 years (AOR = 2.09, 95% CI: 1.10–3.97, *p* = 0.024). Compared with participants with normal BMI, those who were overweight (AOR = 1.82, 95% CI: 1.03–3.20, *p* = 0.037) or obese (AOR = 2.46, 95% CI: 1.24–4.88, *p* = 0.010) had significantly higher adjusted odds of hypertension. Abdominal obesity was also significantly associated with higher odds of hypertension (AOR = 2.57, 95% CI: 1.41–4.65, *p* = 0.002). No statistically significant associations were observed for sex, education level, dyslipidemia, individual lipid parameters (TG, TC, HDL‐C, or LDL‐C), physical activity, or smoking status.

**TABLE 3:** Multivariable logistic regression analysis of factors associated with hypertension among individuals with diabetes.

	Adjusted OR	95% Cl	*p* value
Gender			
Male	Ref		
Female	0.872	(0.37 – 2.03)	0.751
Age groups			
30–40	Ref		
41–55	1.620	(0.94 – 2.78)	0.080
> 55	2.091	(1.10 – 3.97)	0.024
BMI group			
Normal	Ref		
Overweight	1.823	(1.03 – 3.20)	0.037
Obesity	2.463	(1.24 – 4.88)	0.010
WC groups			
Normal	Ref		
Abdominal obesity	2.566	(1.41 – 4.65)	0.002
Education level			
Illiterate	Ref		
Primary	1.888	(0.75 – 4.70)	0.172
Secondary/higher secondary	1.154	(0.51 – 2.60)	0.731
Graduate/above graduate	1.011	(0.45 – 2.26)	0.979
Dyslipidemia			
No	Ref		
Yes	1.636	(0.59 – 4.51)	0.342
TG			
Normal	Ref		
Elevated	0.818	(0.48 – 1.37)	0.448
TC			
Normal	Ref		
Elevated	0.976	(0.55 – 1.71)	0.932
HDL‐C			
Normal	Ref		
Low	0.739	(0.38 – 1.42)	0.368
LDL‐C			
Normal	Ref		
Elevated	1.079	(0.61 – 1.90)	0.793
Physical activity			
Low	Ref		
Medium	0.575	(0.29 – 1.12)	0.104
Adequate	0.683	(0.24 – 1.93)	0.474
Smoking			
No	Ref		
Yes	0.590	(0.32 – 1.06)	0.080

*Note:* Ref, reference category. AORs were estimated using multivariable logistic regression with hypertension as the dependent variable. Percentages are based on the available data for each variable.

Abbreviations: AOR, adjusted odds ratio; CI, confidence interval.

## 4. Discussion

In this study, the prevalence of hypertension was high at 61.4%. This rate is comparatively higher than that of the general adult population in Bangladesh, where hypertension varies between 25% and 35% depending on participant’s age and study area [18]. The higher rate in diabetic patients indicates the known relationship between these two chronic conditions. Diabetes accelerates vascular aging, causes endothelial dysfunction, and increases arterial stiffness, all of which raise blood pressure [19].

The prevalence and factors identified in this study are consistent with those from other areas of Bangladesh and nearby South Asian countries. For example, similar studies in Afghanistan, Bangladesh, and India have reported hypertension prevalence from 55% to 67% among diabetic individuals, with increased age and obesity being the main factors associated with this condition [20, 21].

Age was identified as a significant determinant of hypertension. Participants over 55 years of age were about twice as likely to have hypertension compared to those aged 30–40 years. This finding aligns with earlier studies in Bangladesh and other low‐ and middle‐income countries, which consistently show that the risk of hypertension increases with age among diabetic population [9]. Physiologically, aging causes arterial wall thickening, reduces elasticity, and reduces sensitivity to blood pressure changes. Together, these changes raise systolic blood pressure [22]. Furthermore, prolonged diabetes among older individuals causes vascular and renal complications, which may also predispose them to hypertension [22].

Both general and abdominal obesity were significant predictors of hypertension among the participants. Obese participants had about 2.5 times higher odds of hypertension compared to those with a normal BMI. Abdominally obese individuals had 2.6 times greater odds. These findings correspond with regional and global evidence showing a strong relationship between adiposity and hypertension in both diabetic and nondiabetic populations [23].

The biological mechanisms linking obesity and high blood pressure are complex. Adipose tissue raises the activity of the sympathetic nervous system, increases sodium retention through the renin–angiotensin–aldosterone system, and increases systemic inflammation [24]. Abdominal obesity, especially, causes more visceral fat accumulation, which produces adipokines and inflammatory cytokines that affect the vascular endothelial function [25]. In diabetic individuals, these processes become even worse due to insulin resistance and oxidative stress which increases the risk of hypertension [2].

In this study, gender, education, and physical activity did not show a significant association with hypertension. Most cases occurred in males due to a larger sample size; however, this gender difference was not statistically significant [7, 26]. This aligns with national data showing mixed trends, as some studies in Bangladesh indicate a higher prevalence in women, while others report similar rates across both sexes [7, 26]. The limited number of female participants may limit the detection of meaningful differences. Physical activity levels also showed no strong relationship with hypertension, and this might be due to self‐reported data or minimal variation in activity levels. Previous studies suggest that moderate‐to‐vigorous physical activity can significantly lower hypertension risk in diabetics by improving insulin sensitivity, vascular function, and weight management [27].

No significant association was observed between lipid parameters and hypertension. Although dyslipidemia and hypertension are both parts of metabolic syndrome, their direct cross‐sectional relationship is often inconsistent [28, 29]. One reason might be that most participants were already on diabetes management and possibly taking medications to lower lipids, which may have reduced the observed differences. Smoking also showed a nonsignificant association with hypertension, which might be a reason that only 19% of the participants reported smoking which limited the association.

This study’s findings are significant for managing diabetes in Bangladesh. Having both diabetes and hypertension greatly raises the risk of cardiovascular disease, kidney, and cerebrovascular complications. Therefore, screening for hypertension should be part of regular diabetes care, particularly for older and overweight people. Lifestyle changes, including dietary advice, encouraging physical activity, and reducing weight, should be a focus in both community and clinical settings.

This study has several limitations. First, the cross‐sectional design prevents establishing temporal or causal relationships between the identified factors and hypertension. Second, participants were recruited from selected areas of the Sylhet region, which may introduce selection bias and limit the generalizability of the findings to the broader Bangladeshi population. Third, the study population was significantly skewed by sex, with women representing only 8.1% of participants. Although both sexes were invited using the same recruitment procedures, substantially fewer women volunteered to participate. Consequently, sex‐specific comparisons had limited statistical power and should be interpreted cautiously. Fourth, some variables had missing observations, which may have introduced bias and reduced the effective sample size for specific analyses. Fifth, although several clinically relevant covariates were included in the multivariable logistic regression model, residual confounding cannot be excluded. Potentially important factors, including diabetes duration and treatment, antihypertensive medication use, dietary sodium intake, socioeconomic status, family history of hypertension, and other lifestyle or clinical factors, were not fully assessed or adjusted for. Sixth, the use of nonprobability convenience sampling may have caused selection bias as individuals who participated might differ systematically from those who were not recruited or declined to take part. As a result, the findings may not be fully generalizable to all individuals with diabetes in the Sylhet region or in Bangladesh. Finally, self‐reported information on some behavioral factors may be subject to recall or reporting bias. Therefore, the observed associations should be interpreted as associations rather than causal effects. Future prospective, multicenter studies with more representative and sex‐balanced samples and comprehensive assessment of potential confounders are warranted.

## 5. Conclusion

This study demonstrated a high prevalence of hypertension among adults with diabetes in the Sylhet region of Bangladesh. Older age, general obesity, and abdominal obesity were independently associated with hypertension. These findings highlight the importance of integrating routine blood pressure screening, weight management, and lifestyle interventions into diabetes care programs to reduce the risk of cardiovascular and renal complications. Public health strategies that promote healthy dietary habits, regular physical activity, and obesity prevention may further contribute to reducing the burden of hypertension among individuals with diabetes. Given the cross‐sectional design, the observed associations should not be interpreted as causal. Future prospective studies are warranted to confirm these findings and evaluate the effectiveness of integrated management strategies in improving cardiometabolic health outcomes.

## Author Contributions

Nurshad Ali conceived and designed the study, interpreted the data, and wrote the manuscript. Abu Taher conducted the experiments, performed the data analysis, and contributed to drafting the manuscript.

## Funding

This study did not receive any external funding.

## Disclosure

All authors critically reviewed the manuscript, approved the final version, and agreed to be accountable for all aspects of the work.

## Conflicts of Interest

The authors declare no conflicts of interest.

## Data Availability

The data are available from the corresponding author upon reasonable request.
